# Eosinophil recruitment and activation: the role of lipid mediators

**DOI:** 10.3389/fphar.2013.00027

**Published:** 2013-03-22

**Authors:** Tatiana Luna-Gomes, Patrícia T. Bozza, Christianne Bandeira-Melo

**Affiliations:** ^1^Instituto de Biofïsica Carlos Chagas Filho, Universidade Federal do Rio de JaneiroRio de Janeiro, Brazil; ^2^Instituto Oswaldo CruzRio de Janeiro, Brazil

**Keywords:** eosinophil, chemotaxis, lipid mediators, prostaglandins, leukotrienes

## Abstract

Eosinophils are effector cells that migrate toward several mediators released at inflammatory sites to perform their multiple functions. The mechanisms driving eosinophil selective accumulation in sites of allergic inflammation are well-established and involve several steps controlled by adhesion molecules, priming agents, chemotactic, and surviving factors. Even though the majority of studies focused on role of protein mediators like IL-5 and eotaxins, lipid mediators also participate in eosinophil recruitment and activation. Among the lipid mediators with distinguish eosinophil recruitment and activation capabilities are platelet activating factor and the eicosanoids, including leukotriene B_4_, cysteinyl leukotrienes, and prostaglandin D_2_. In this review, we focused on the role of these four lipid mediators in eosinophil recruitment and activation, since they are recognized as key mediators of eosinophilic inflammatory responses.

Eosinophils are nowadays considered as multifunctional cells that have long been associated with allergy and parasitic infections. They are immunomodulatory cells that participate both in innate and adaptive immune response via expression of various receptors and secretion of a variety of mediators. To perform their functional activities, first eosinophils must migrate to sites of inflammatory reaction. Over the last years, a number of mediators and receptors involved in the regulation of eosinophil recruitment have been identified. Besides adhesion molecules and cytokines, eosinophil mobilization is mostly coordinated by a broad range of bioactive mediators known as chemokines. These molecules are an increasing family of small proteins with common structural motifs that via activation of their specific receptors play an important role not only in selective recruitment of eosinophils but also in subsequent eosinophil activation in sites of eosinophilic inflammation. Even though the main efforts in this research area are directed toward peptidic mediators, like chemokines, a growing body of data has unveiled key roles of lipid mediators in regulating eosinophil migration and activation. Among bioactive lipids, eicosanoids are a large family of distinctive mediators derived from arachidonic acid (AA) metabolization regularly found at high levels in inflammatory sites. Displaying from pro- to anti-inflammatory, pro-resolution, and even immunomodulatory functions, these molecules are key mediators in the pathogenesis of diverse inflammatory diseases, such as asthma, infection, and cancer. This review will first explore the role of some of the most well-studied lipid mediators on eosinophil migration. Then, it will summarize the impact of a varied of these mediators on eosinophil activation, focusing on eosinophil secretory function of leukotriene C_4_ (LTC_4_) synthesis/release.

## HOW DO LIPID MEDIATORS IMPACT EOSINOPHIL MIGRATION?

Eosinophilia is a classical feature of allergic inflammatory responses, therefore regulation of eosinophil migration to the inflammatory focus is a critical stage in the processes of chronic inflammation that affect, for instance, asthmatic airways. Eosinophil recruitment into the tissues after immune or chemical stimuli requires the production of chemoattractants by several cells such as macrophages, mast cells, or lymphocytes. Briefly, local increase in the secretion of eosinophilotactic molecules, leads to eosinophil adhesion to the endothelium through interaction with selectins expressed on the vascular endothelium followed by firm adhesion through interaction with integrins. Subsequent transmigration through the endothelial cell monolayer is followed by chemotaxis in the tissue, a process known to be largely controlled by chemokines such eotaxina-1, 2, 3, and RANTES and their specific receptors, especially CCR3 (Simson and Foster, 2000). However, both *in* *vivo* and *in vitro*, eosinophils also migrate toward different factors distinct from chemokines such as C5a (Klebanoff et al., 1977), interleukin-5 (IL-5; Wang et al., 1989), granulocyte-macrophage colony-stimulating factor (GM-CSF; Secor et al., 1990), and lipid mediators. Indeed, AA metabolites as leukotrienes and prostaglandins (PGs), as well as, platelet activating factor (PAF) are considered major players in the pathogenesis of asthma and other forms of allergic inflammation, in part because they control eosinophil influx and activation.

Within a variety of cell types, phospholipase A_2_-driven AA mobilization followed by the oxidative metabolism of free AA mediated by either two cyclooxygenases (COX, PG H synthase) or a family of lipoxygenase (LO) enzymes culminate with the generation of bioactive lipid mediators with roles in eosinophilic inflammation. Specifically concerning those with ability to elicit eosinophil recruitment, newly synthesized lipid mediators may comprise:

### LEUKOTRIENE B_4_

**FIGURE 1:**
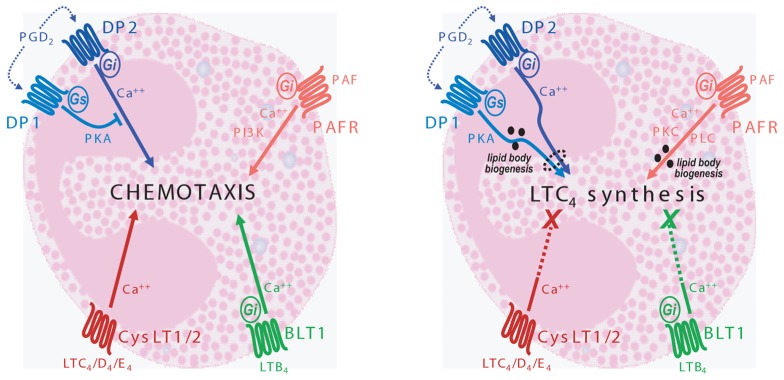
**Schematic mechanisms of LTB_4_-, LTC_4_-, PAF-, or PGD_2_-induced eosinophil chemotaxis and LTC_4_ synthesis**. Left eosinophil scheme displays the ability of the four lipid mediators to trigger eosinophil chemotaxis by activating receptor-mediated distinct intracellular signaling. In contrast, the right eosinophil scheme shows that only PGD_2_ and PAF are capable of activating LTC_4_ synthesizing machinery, yet again by eliciting distinct signaling, but both by a lipid body-dependent mechanism. The right scheme also illustrates that both leukotrienes LTB_4_ and LTC_4_, even thought activate their specific receptors in eosinophils (see left panel), failed to trigger lipid body biogenesis or LTC_4_ synthesis.

Leukotriene B_4_ (LTB_4_) is a lipid mediator with potent chemoattractant properties that is rapidly generated from activated innate immune cells such as neutrophils, macrophages, and mast cells. Elevated levels of LTB_4_ have been reported in various allergic diseases and these levels have been related to disease activity and eosinophilia (O’Driscoll et al., 1984; Wardlaw et al., 1989; Shindo et al., 1993). LTB_4_ can bind to two highly conserved G protein-coupled receptors (GPCRs), LTB_4_ receptor 1 (BLT1) and the considered low-affinity BLT2 (Toda et al., 2002; Yokomizo, 2011). LTB_4_ serves as a potent chemoattractant through ligation of BLT1 on target cells. Expression and function of LTB_4_ receptors on eosinophils remained for long time controversial, in part because LTB_4_-driven activity seemed to have some selectivity toward neutrophils. However, while strong demonstration of BLT1 expression in human eosinophils is still pending, functional assays using LTB_4_ as agonist and specific BLT1 antagonists have provided evidences of expression of active BLT1 on human eosinophils. For instance, it has been shown a BLT1-driven LTB_4_ ability to trigger calcium influx in human eosinophils (Murray et al., 2003a). On the other hand, murine (m)BLTR was cloned while searching for novel chemoattractant receptors in murine eosinophils and demonstrated that it encodes a functional receptor for LTB_4_ which are able to trigger chemotaxis of mouse eosinophils (**Figure 1**, left panel; Spada et al., 1997; Huang et al., 1998). Reinforcing both *in vitro* data and *in vivo* assays with BLT1 antagonists, *in vivo* studies using BLT1-deficient mice have confirmed that ligation of BLT1 by LTB_4_ is a key event for recruitment of eosinophils (Tager et al., 2000) However, it is noteworthy that while mouse eosinophils may generate only negligible amounts of LTB_4_, human eosinophils are not LTB_4_ producers, representing major cellular sources of cysteinyl LTs (Weller et al., 1983). Based on the prominent eosinophil feature of recurrently depend on autocrine/paracrine stimulation to regulate their own functions, it seemd to be potentially more important the role of cysteinyl LTs in inducing eosinophilic responses, including autocrine/paracrine roles in induction of eosinophil chemotaxis and activation.

### CYSTEINYL LEUKOTRIENES

Leukotriene C_4_ and its extracellular derivatives LTD_4_ and LTE_4_ have many well recognized actions as mediators of allergic response, causing bronchoconstriction, mucous hypersecretion, increased microvascular permeability, and bronchial hyperresponsiveness. Additional but not as well-established effect is the ability of cysteinyl LTs to control eosinophil activities, including those related to tissue infiltration. Involvement of cysteinyl LTs in eosinophil influx is an *in vivo* phenomenon which was firstly demonstrated in guinea-pigs (Chan et al., 1990), but also observed in human (Laitinen et al., 1993) and reinforced by the anti-allergic effects of CysLT1 antagonists which, in addition to inhibiting allergic symptoms, also inhibit eosinophil recruitment during airway allergic inflammation (Peters-Golden, 2008). Even though cysteinyl LTs display negligible eosinophilotactic activity *in vitro* (**Figure 1**, left panel; Fregonese et al., 2002), cysteinyl LTs contribute to several mechanisms involved in mouting tissue eosinophilia, since: (i) cysteinyl receptor CysLT1 appears to play a role in eosinophilopoeisis, inasmuch as CysLT1 antagonism *in vivo* limits IL-5-responsive eosinophil differentiation and maturation (Saito et al., 2004); (ii) cysteinyl LTs are able to significantly up-regulate adhesion molecules, such as Mac-1 expression (Fregonese et al., 2002; Saito et al., 2004); (iii) direct administration of LTC_4_ induce a rapid and significant reduction in leukocyte rolling velocity, further increasing cell adherence odds (Kanwar et al., 1995); (iv) cysteinyl LTs induce RANTES production from isolated lung cells, which in turn might cause RANTES-driven migration of eosinophils into airways (Kawano et al., 2003).

### PLATELET ACTIVATING FACTOR

One major chemoattractant for eosinophils is the ether-linked phospholipid, PAF. PAF (1-*O*-alkyl-2-acetyl-*sn*-glycero-3-phosphocholine) is another potent lipid mediator synthesized by a range of cell types, including monocytes/macrophages, mast cells, platelets, neutrophils, endothelial cells as well as eosinophils. PAF is capable of eliciting both chemokinesis and chemotaxis *in vitro* and triggering eosinophil influx and accumulation *in vivo* (Wardlaw et al., 1986; Kimani et al., 1988; Martins et al., 1989; Kato et al., 2004). Acting via a single class of identified receptor – named PAFR – a seven–*trans*-membrane G protein-coupled receptor, PAF evokes not only migration-related activities but also a variety of eosinophilic functional responses (Grigg, 2012). Of note, while it became more and more clear that human and mouse eosinophils shared profound dissimilarities (Lee et al., 2012), both express functional active PAFR which mediates eosinophilotactic activity of PAF in human and mouse cells by a pertussis toxin (PTX)-sensitive manner. Several studies have tryed to characterize the signaling pathways involved in PAF-induced eosinophil chemotaxis, and although still controversial, it is now recognized that eosinophilotactic responses triggered by PAF depend on activation of mitogen-activated protein (MAP) kinases, while upstream signaling events are regulated by activation of phosphoinositide 3-kinase (PI3K; **Figure 1**, left panel; Dent et al., 2000; Miike et al., 2000). Indeed, these findings are in agreement with the demonstration that PI3K inhibitors suppress PAF-mediated tissue eosinophilia in diseases such as asthma (Mishra et al., 2005).

### PROSTAGLANDIN D_2_

Prostaglandin D_2_ has emerged as a key mediator of allergic diseases such as asthma (Matsuoka et al., 2000), in part due to its now well-characterized ability to promote potent eosinophil chemotaxis and activation (Powell, 2003). PGD_2_-driven cellular functions are all mediated by high-affinity interaction with two receptors, namely D prostanoid receptor 1 (DP_1_) and chemoattractant receptor-homologous molecule expressed on T helper type 2 cell (Th2) cells (CRTh2, also known as DP_2_). Whilst DP_1_ is coupled to Gαs protein and signals through elevation of intracellular levels of cyclic adenosine monophosphate (cAMP), DP_2_ is coupled to Gαi and its activation leads to elevation of intracellular calcium, reduction in cAMP (Sawyer et al., 2002) and downstream activation of PI3K (Xue et al., 2007). Eosinophils co-express both the classic DP_1_ receptors coupled to adenylyl cyclase, as well as, PTX-sensitive DP_2_ (Monneret et al., 2001).

Prostaglandin D_2_-mediated eosinophilotactic effect is due to direct activation of the DP_2_ receptor expressed on eosinophil surface (Monneret et al., 2003). Several pharmacological studies show the involvement of DP_2_ in the establishment of eosinophilia in models of allergic inflammation. For instance, intratracheal injection of PGD_2_ or selective DP_2_ agonist induced eosinophilia in rats, whereas the use of selective DP_1_ agonist failed to trigger eosinophil accumulation (Emery et al., 1989). Likewise, intratracheal administration of DP_2_ agonist or PGD_2_ induced specific airway eosinophilia in mice previously exposed to the allergen or IL-5 (Shiraishi et al., 2005). DP_2_ antagonist abrogated the PGD_2_-induced mobilization of eosinophils from the bone marrow of the guinea-pig confirming a crucial role of DP_2_ in this response (Royer et al., 2008). A specific DP_2_ agonist not only increased eosinophil recruitment at inflammatory sites but also the pathology in two *in vivo* models of allergic inflammation: atopic dermatitis and allergic asthma (Spik et al., 2005). Concurring, selective DP_2_, but not DP_1_ antagonists were capable to inhibit eosinophil accumulation in a model of PGD_2_-induced eosinophilic pleurisy (Mesquita-Santos et al., 2011). *In vitro*, PGD_2_ is able to promote additional migration-related activities, such as increased expression of cell adhesion molecules CD11b and L-selectin, calcium mobilization, actin polymerization, chemokinesis and a rapid change in eosinophil morphology (Gervais et al., 2001; Monneret et al., 2001). Of note and as illustrated in **Figure 1** (left panel), these and other *in vitro* studies have collectively unveiled that PGD_2_-driven eosinophil chemotaxis may be determined by a balance between opposing downstream signaling pathways: cAMP-dependent inhibitory DP_1_ versus prevailing stimulatory DP_2_ intracellular effects (Monneret et al., 2003; Ulven and Kostenis, 2006; Sandig et al., 2007). However, further studies appears to be still needed to fully explain PGD_2_ mechanisms of actions, since recently it has been shown that DP_1 _and DP_2 _may form heteromers representing a distinct functional signaling unit on eosinophil membrane with non-changed ligand-binding features (Sedej et al., 2012). In fact, these are not the first findings showing the ability of DP_1_ receptors to amplify the biological response to DP_2_ activation in eosinophils (Mesquita-Santos et al., 2011) a process that, although may not play roles in eosinophil migration, it appears to be critical to PGD_2_-induced eosinophil activation (see below).

## DO LIPID MEDIATORS ACTIVATE EOSINOPHIL EFFECTOR FUNCTIONS?

At the sites of eosinophilic accumulation, through their ability to secrete a range of cytokines, basic proteins, reactive oxygen species as well as lipid mediators, eosinophils contribute to the physiopathology of a growing list of conditions including classical eosinophil-related diseases such as bronchial asthma, novel and quite surprising pathologies such as cancer, multiple sclerosis, Duchene muscular dystrophy as well as physiological process such as mammary development (Jacobsen et al., 2012). While the regulation of eosinophil migration to the inflammatory focus is a critical stage in eosinophilic pathologies, understanding the mechanisms by which eosinophil activation is stimulated and its consequences appear to be even more important in defining potential targets for therapeutic interventions, since the specific stimulatory molecules, its receptors and signaling pathways involved in eosinophil activation and subsequent mediator secretion may each be susceptible to inhibition. Indeed among different parameters of eosinophil activation, eosinophil secretory activity may represent the most attractive target to development of therapeutical maneuvers. Upon activation, eosinophil may engage both in secretion of pre-formed granule-stored contents, including eosinophil specific toxic proteins, enzymes, cytokines, chemokines, and other bioactive mediators, as well as *de novo* synthesized/released molecules including oxygen free radicals but prominently lipidic AA-derived mediators. The unique eosinophil pattern of oxidative metabolism of AA generates a specific array of eicosanoids. Eosinophils can synthesize lipoxin A_4_ (LXA_4_) and the aptly named after eosinophils, eoxin C_4 _(EXC_4_), besides the prostanoids thromboxane B_2_ (TXB_2_), PGE_2_ and the recently identified PGD_2_. However, when properly stimulated, eosinophils prominently synthesize cysteinyl LTs. Of note, eosinophils are a major cellular source of cysteinyl LTs and have been identified as the principal LTC_4_ synthase expressing cells in bronchial mucosal biopsies of asthmatic subjects (Bandeira-Melo and Weller, 2003). Hence, much interest in understanding the regulation of eicosanoid formation in eosinophils has focused on the mechanisms that regulate eosinophil cysteinyl LTs formation and release. Briefly, free AA can be metabolized within eosinophils by 5-LO, which is the limiting enzyme of leukotriene synthesis. 5-LO catalyzes a two-step reaction. First, 5-LO targets free AA in concert with the 5-LO-activating protein (FLAP) to insert one oxygen molecule into the 5 position of AA to form 5S-hydroperoxyeicosatetraenoic acid (HPETE), then transforms 5-HPETE into an unstable allylic epoxide, named LTA_4_. The subsequent metabolism of LTA_4_ also differs between leukocytes. In neutrophils, for instance, LTA_4_ hydrolase enzymatically hydrolyses 5-LO-metabolite LTA_4_ to LTB_4_. In contrast within human eosinophils, which do not express LTA_4_ hydrolase and therefore are incapable of LTB_4_ synthesis, a specific glutathione S-transferase, named LTC_4_ synthase (LTC_4_S), catalyzes the adduction of reduced glutathione (a tripeptide composed by glutamic acid, glycine, and cysteine) to LTA_4_ to form LTC_4_. After energy-dependent export, LTC_4_ is converted to LTD_4_ and LTE_4_ through sequential enzymatic removal of the glutamic acid by γ-glutamyl transpeptidases and then the glycine by dipeptidases. Therefore, because these LTs share a cysteine, LTC_4_ and its extracellular derivatives LTD_4_ and LTE_4_ are collectively called cysteinyl LTs.

Similar to how we presented the roles of lipid mediators in inducing eosinophil migration, here we will also summarize some activating roles of LTB_4_, cysteinyl LTs, PAF and PGD_2_, but we will give special emphasis to a prototype parameter of eosinophil activation: eosinophil ability to activate LTC_4_ synthesizing machinery.

### LEUKOTRIENE B_4_

Even though LTB_4_ receptors have been indirectly and directly found to be expressed on human and murine eosinophils, respectively, there are not many successful studies reporting LTB_4_-driven eosinophil activation. Mainly using as cell model guinea-pig eosinophils, it has been shown that LTB_4_ was capable of stimulating eosinophil recruitment, release of AA, homotypic eosinophil aggregation, as well as, rapid and transient activation of the NADPH oxidase (Faccioli et al., 1991; Lindsay and Giembycz, 1997; Teixeira et al., 1999). Of note, the intracellular mechanisms that mediate LTB_4_-induced NADPH oxidase activation involve mediation by lyn kinase, PKC, and PLA_2_, but occurs essentially independently of changes in the intracellular calcium, phospholipase D, PI3K, and ERK1/2 (Perkins et al., 1995; Lindsay et al., 1998a,b; Lynch et al., 1999) Specifically regarding induction of LTC_4_ synthesizing function, stimulation of human eosinophils with LTB_4_ failed to mount a LTC_4 _ synthesizing response (**Figure 1**, right panel). In addition, eosinophil stimulation with LTB_4_ was also unable to trigger synthesis of other eicosanoids such as PGE_2_ or even the biogenesis of lipid bodies – organelles, which compartmentilize AA metabolism within eosinophils and other cell types, and that are promptly assembled under stimulation that leads to eicosanoid synthesis (Bozza et al., 1997b).

### PLATELET ACTIVATING FACTOR

Human eosinophils are prominent among cell populations that respond to PAF stimulation displaying, besides chemotaxis, numerous PAF-driven functions, including migration-related activities such as adhesion and expression of cell surface molecules, as well as, secretory functions, including superoxide production and release of cationic granule proteins and stored cytokines (Wardlaw et al., 1986; Kroegel et al., 1989; Zoratti et al., 1991; Takizawa et al., 2002; Dyer et al., 2010). Equally important is the notion that although only one PAFR has been identified, PAF-driven signaling has emerged as a complex phenomenon, displaying differences between eosinophil chemotactic versus secretory functions and therefore suggesting the existence of yet non-characterized receptors (Kato et al., 2004).

It is noteworthy that PAF was the first stimulus to have its lipid body-dependent mechanism of eliciting LTC_4_ synthesis characterized. PAF, acting via its G-protein-linked receptor induces lipid body formation via a downstream signaling involving PKC and phospholipase C (PLC) activation (**Figure 1**, right panel; Bozza et al., 1996, 1997a, 1998). Even more relevant to PAF ability of inducing LTC_4_ synthesis, it was the demonstration that the major enzymes involved in the enzymatic conversion of AA into LTC_4_, 5-LO, and LTC_4_ synthase, were found compartmentalized within PAF-induced newly assembled eosinophil lipid bodies (Bozza et al., 1997a, 1998) and that these enzymes were functional and producing LTC_4 _ within theses organelles (Bandeira-Melo et al., 2001).

### CYSTEINYL LEUKOTRIENES

Cysteinyl leukotrienes exert their actions by engaging specific receptors. Al least two cysLT receptors (cysLTRs) have been cloned and characterized, the CysLT1 and CysLT2 receptors (Lynch et al., 1999; Sarau et al., 1999; Heise et al., 2000; Nothacker et al., 2000). These receptors can be distinguished with pharmacologic inhibitors and by their differing ligand-binding affinities. In addition, various findings suggest the existence of other, not yet cloned, cysLTR (Panettieri et al., 1998; Ravasi et al., 2000; Mellor et al., 2002).

Inasmuch as eosinophils express functional receptors for cysteinyl LTs, it has been investigated their potential role as stimuli of eosinophil activation. Indeed, a series of reports showed cysteinyl LTs ability to affect various eosinophil responses. For instance, cysteinyl LTs promote CysLT_1_-dependent calcium influx on HL-60 (Thivierge et al., 2000; Murray et al., 2003b). We have also shown that LTC_4_, LTD_4_, and LTE_4_ induced a dose- and time-dependent, vesicular transport-mediated release of pre-formed IL-4 from eosinophils derived *in vitro* from human cord blood progenitors (Bandeira-Melo et al., 2002a). Although some controversy exist (Murray et al., 2003b), cysteinyl LTs also appear to be able to induce *in vitro* survival of human eosinophils by activation of CysLT_1_ receptors (Lee et al., 2000; Becler et al., 2002). It is noteworthy that in addition to their recognized activities as paracrine mediators, eicosanoids like cysteinyl LTs are now also recognized to display autocrine effects. Indeed, eosinophil-derived cysteinyl LTs exert autocrine effects to enhance eosinophil survival triggered by GM-CSF, as well as, mast cell- and lymphocyte-derived molecules (Lee et al., 2000). Moreover, the capacity of eotaxin to stimulate the vesicular transport-mediated release of pre-formed IL-4 from human eosinophil granules is dependent of an endogenous LTC_4_, formed at eosinophil lipid bodies, that acting as an intracrine signaling molecule regulates this CCR3-elicited IL-4 release (Bandeira-Melo et al., 2002c). Thus, LTC_4_ may act intracellularly as intracrine signal transducing mediators. Indeed, cysteinyl LTs-responsive receptors have been identified on the membranes of intracellular eosinophil granule organelles and appear to function mediating cysteinyl LTs-stimulated secretion from within eosinophil granules, including those granules found extracellularly (Neves et al., 2010). On the other hand ans as illustrated in **Figure 1** (right panel), specifically regarding the ability of activating LTC_4_ synthesis, none endogenous or exogenous cysteinyl LTs displayed the ability to trigger lipid body biogenesis or to elicit their own synthesis (Bandeira-Melo et al., 2002c).

### PROSTAGLANDIN D_2_

Besides migration-related cell functions, it is now well-characterized that PGD_2_ is a potent inducer of eosinophil activation, being capable of promoting eosinophil secretory activity. For instance, PGD_2_ is capable of triggering eosinophil degranulation, which appears to be induced by the selective DP_2_ agonist but not by selective DP_1_ agonist, suggesting for DP_2_ a role in modulating, not only eosinophil migration, but also activation (Gervais et al., 2001). We have also shown that, in addition to its eosinophilotactic activity, PGD_2_ controls allergy-relevant eosinophil activation parameter: the increased LTC_4_-synthesizing capacity of these cells (Mesquita-Santos et al., 2006). Indeed, other eosinophilotactic mediators, including eotaxin, RANTES, and PAF are capable of triggering LTC_4_ synthesis within eosinophils through activation of their cognate Gαi-coupled chemotactic receptors (e.g., CCR3; Bozza et al., 1996; Bandeira-Melo et al., 2001). However, PGD_2_-induced LTC_4_ synthesis, surprisingly and distinctly from other parameters of eosinophil activation evoked by PGD_2_, was not mediated by the stimulatory activation of DP_2_ receptors while being counter-balanced by a parallel inhibitory cAMP-dependent DP_1_ receptor activation. On contrary, it does depend on a novel kind of interaction between the PGD_2_ receptor types expressed on eosinophils (**Figure 1**, right panel). Eosinophil LTC_4_ synthesis triggered by PGD_2_ is controlled by complementary stimulatory events between DP_1_ receptor-activated lipid bodies and concurrent DP_2_ receptor signaling (Mesquita-Santos et al., 2011). While PGD_2_ emerges as a potent inflammatory mediator of allergic disorders and as an interesting therapeutic target, because of the mandatory dual activation of DP_1_ and DP_2_ receptors for increasing eosinophil LTC_4_ synthesis, either DP_1_ or DP_2_ receptor antagonists might be highly effective candidates as anti-allergic tools to control cysteinyl LTs production regulated by the activation of eosinophils at sites of allergic reactions. On the top of that, we had recently also found out that upon proper stimulation, both human and mouse eosinophils can produce significant amounts of biologically relevant PGD_2_ (Luna-Gomes et al., 2011)_._ PGD_2_ intracellular synthesis within eosinophils led to PGD_2_ receptor-mediated paracrine/autocrine functions, contributing to eosinophil activation. Indeed, eosinophil-derived PGD_2_ appears to be capable of regulating both eosinophil motility, as well as, lipid body-driven LTC_4_ synthesis within eosinophils stimulated with eotaxin, for instance.

## FINAL REMARKS

It is clear that several relevant aspects of lipid mediator impact on eosinophil biology need to be further characterized, however knowledge on this subject had evolved dramatically in the last decades. Among the most significant advances on eosinophil/lipid mediator axis are: (i) the recognition that eosinophils express the multitude of lipid mediator receptors on their surface, even those receptor pairs with apparently opposing functional outcomes under activation; (ii) the appreciation that not only eosinophil migration is elicited by lipid mediators, but maybe even more therapeutically relevant, activation of eosinophil secretory functions; and (iii) the acknowledgment of a wide-ranging induced signaling and consequently functional potentiality for lipid mediator-stimulated eosinophils that have still unpredicted impact to surrounding eosinophilic immuno-pathologies.

Still of special interest for eosinophil biology with roles in maximizing eosinophil functional potentialities is the rising observations unraveling intricate interactions between lipid mediators (such as LTC_4_ and PGD_2_) and eosinophil-relevant chemokines and other proteic stimuli. Possibly the most illustrative example of such cross-talking is eosinophil stimulation by eotaxin, a key mediator in the development of allergic eosinophilia that is known by its potent eosinophilotactic activity and has emerged as a potent mediator of eosinophil activation. Among a number of data on eotaxin/AA metabolites interdependency, some hallmarks are the sequencial events: (i) eotaxin particular ability to acutely enhance PGD_2_ synthesis by eosinophils by stimulating CCR3 receptors (Mesquita-Santos et al., 2006; Luna-Gomes et al., 2011); (ii) the subsequent autocrine/paracrine induction of lipid body biogenesis and lipid body-located LTC_4_ synthesis by eosinophil-derived PGD_2_ (Luna-Gomes et al., 2011); followed by (iii) LTC_4_-driven intracrine induction of piecemeal degranulation of granule-stored IL-4 by eotaxin-stimulated eosinophils (Bandeira-Melo et al., 2002c). Nevertheless, eotaxin is not the only example of such lipid/protein cooperation. It is still noteworthy that cell types other than eosinophils also undergo such lipid mediator/protein mediator cross-talking in regulating cell activation. Either infection-elicited or oxLDL-driven MCP1, for instance, besides its known CCR2-driven chemotactic function, appear as a key activator of lipid body biogenic and leukotriene synthesizing machineries within macrophagic cells (Pacheco et al., 2007; Silva et al., 2009). Once more specifically regarding eosinophils, synergistic effects on eliciting eosinophil chemotaxis have been also described between PGD_2_ and at least the cytokines IFN-γ and TNF-α (El-Shazly et al., 2011), as well as, between DP_2_ activation and vasoactive intestinal peptide VIP (El-Shazly et al., 2013). Moreover, RANTES, IL-16 and MIF are also proteic mediators capable of activating eicosanoid synthesizing machinery within eosinophils culminating with the generation of LTC_4_ and PGD_2_, that in turn intracrinally or autocrinally mediate eosinophil secretory functions (Bandeira-Melo et al., 2002b,c; Vieira-de-Abreu et al., 2011).

## Conflict of Interest Statement

The authors declare that the research was conducted in the absence of any commercial or financial relationships that could be construed as a potential conflict of interest.
